# The role of the gut microbiome in the intergenerational transmission of the obesity phenotype: A narrative review

**DOI:** 10.3389/fmed.2022.1057424

**Published:** 2022-12-22

**Authors:** Mabel Tang, Elisa Marroquin

**Affiliations:** ^1^Department of BioSciences, Rice University, Houston, TX, United States; ^2^Department of Nutritional Sciences, Texas Christian University, Fort Worth, TX, United States

**Keywords:** gut microbiota, maternal obesity, pregnancy, cesarean delivery, breastfeeding, probiotic

## Abstract

Obesity is considered an epidemic by the World Health Organization. In particular, maternal obesity can affect the development of obesity and other related metabolic disorders in infants. Recently, both animal and human studies have pointed to the importance of the gut microbiome in facilitating the transmission of the obesity phenotype from mother to offspring. The gut microbiome changes significantly during the progression of pregnancy, and the microbiota of the amniotic fluid and placenta have recently been shown to colonize the infant gut *in utero*. Microbial composition, diversity, and richness are significantly altered by maternal obesity, which in turn affects the infant’s acquisition of the gut microbiome and their risk to develop metabolic disorders. C-section has also been shown to affect the colonization of the infant gut and offspring metabolic and immune health. This narrative review seeks to discuss the role of the gut microbiome in the transmission of the obesity phenotype from mother to child, as well as how birth delivery, breastfeeding, and probiotic interventions may modulate this relationship.

## 1 Introduction

Approximately 40% of women in the US had obesity (1). Maternal obesity can increase the risk of childhood obesity by >2 times (2, 3) which is alarming due to the high obesity levels worldwide (4). Both human and animal models have demonstrated that maternal obesity can contribute to many obesity-linked metabolic and immune disorders in offspring, including increased risk of hypertension, insulin resistance, and systemic inflammation (5, 6). There have been many mechanisms hypothesized to contribute to the intergenerational transmission of obesity, including the previously recognized genetic and environmental factors and the newly identified gut microbial composition.

The gut microbiome of an infant develops significantly during the first year of life and recent animal and human research is trying to explore the role of the gut microbiome in the development of diseases (7, 8). Many human studies have shown that the diversity and composition of the gut microbiome are significantly altered in pregnant women with obesity, which in turn can play a factor in the development of obesity in children (9, 10). For instance, pregnant women with obesity tend to have higher levels of *Bacteroides* and *Staphylococcus* in their third trimester when compared to pregnant women of normal weight (9). Recently, research has established that the maternal gut microbiota exists in the placenta of pregnant women, which strongly supports that the maternal microbiome may be transferred to the infant prior to birth, playing an important role in establishing the infant gut microbiome (11, 12). Thus, there is a need to assess how obesity can affect this transmission and in turn lead to the development of other transgenerational metabolic disorders.

The study of the transmission of the maternal gut microbiota to infants, however, can be complicated by a host of other factors including the method of birth delivery, breastfeeding, and the use of antibiotic and probiotic interventions at an early age (7, 13). Cesarean delivery, or C-section, is a surgical procedure by which an infant is delivered through an incision in the mother’s abdomen, often in the case that vaginal delivery may put the mother or infant at risk. C-section has been shown to alter the gut microbiome in the first year of life, with infants born through C-section having lower *Bifidobacterium*, *Streptococcus*, and *Lactobacillus* genera (2). However, the simultaneous effect of maternal obesity during pregnancy and C-section has not been studied which is represents a key gap in knowledge as mothers with overweight or obesity are more likely to give birth by C-section (14, 15).

Probiotics have been shown to have beneficial effects on the gut microbiota of infants born through C-section, helping it to more closely resemble that of infants born through vaginal delivery (16). However, it is unclear how this effect may differ between babies born to mothers with normal weight versus those born to mothers with obesity.

This review aims to evaluate how the gut microbiome is potentially involved in mother-to-infant obesity phenotype transmission and how this relationship may be modified by the method of birth delivery, breastfeeding, and the use of probiotic treatments. Furthermore, the impact of maternal obesity on their child’s metabolic and immune systems by potentially acting through the passage of gut microbial composition will also be discussed.

## 2 Maternal obesity and pregnancy

### 2.1 Changes in the gut microbiome during pregnancy

To support the development of a growing fetus, the body undergoes many changes that often resemble changes associated with metabolic disorders, including decreased insulin sensitivity and higher levels of inflammatory cytokines (17–19). However, unlike metabolic syndrome, changes in insulin sensitivity and increased adiposity are protective in pregnancy and generally increase nutrient intake for the growing fetus (18, 20). Analysis of the gut microbial composition of pregnant women has shown a significant reduction of phylogenetic diversity from the first to the third trimester (21). Levels of inflammatory cytokines such as IL-2, IL-6, TNF-α, and IFN-γ were also significantly elevated in the stool samples of the third trimester (21). Furthermore, transferring stool samples from women in their third trimester to germ-free mice induced weight gain, decreased insulin sensitivity, and increased inflammation (21). As *Proteobacteria* have previously been associated with inflammation and dysbiosis in human studies, the gut microbiome provides a possible mechanism by which metabolism and immunity are altered during pregnancy (22, 23). Other studies have similarly suggested that the gut microbiome diversity significantly changes throughout pregnancy; however, conflicting evidence exists (24–26). Therefore, more investigation must be completed to elucidate the modifications in the maternal gut microbiome during pregnancy and how these contribute to immune and metabolic changes in both mother and infant.

### 2.2 Effects of maternal obesity on pregnancy-related gut microbiome changes

Pregnant women with obesity have significantly higher levels of *Bacteroides*, *Staphylococcus*, and *Clostridium* (9). *Bacteroides* species have been previously reported to harvest energy more efficiently than other microbes in both human and animal models (9, 27). A significant increase in the relative abundance of *Staphylococcus* and *Enterobacteriaceae* species as well as *Escherichia coli* have also been detected in pregnant women with obesity when compared to pregnant women with normal weight. However, unlike Collado et al., they found a significantly lower level of *Bacteroides* (28). Although the precise relationship between gut microbial composition and weight gain during pregnancy remains to be clarified, it is clear that significant differences in the gut microbial composition of pregnant women with obesity versus pregnant women with a normal BMI do exist.

Short-chain fatty acids (SCFAs) are products of bacterial metabolism in the gut and include acetic acid, butyric acid, and propionic acid. During pregnancy, changes in the gut microbiome result in altered levels of SCFAs. Studies on microbial populations in pregnant women found that women with obesity have significantly reduced levels of butyrate-producing species such as those in the genus *Clostridiales* and *Lachnospiraceae*, and therefore, much lower levels of circulating butyrate (29). Animal models have shown that butyric acid contributes to anti-inflammatory activity by activating regulatory T (Treg) cells and inhibiting IL-17 release (30). Indeed, administration of butyrate to pregnant mice has been demonstrated to reduce levels of pro-inflammatory factors including TNFα and IL1-β (31). Similarly, propionic acid, which is produced by *Bacteroides* and *Firmicutes* species, is reported to have beneficial health effects in both rodents and humans, although the mechanisms of these effects are still relatively unknown (32). On the contrary, propionic acid is positively correlated with HbA1c and plasma glucose levels in pregnant women with obesity (33). Therefore, there is emerging evidence suggesting that changes in the gut microbiome contribute to increased inflammation during pregnancy for women with obesity due to differences in SCFA abundance.

SCFAs have also been reported to have relations with hypertension and type 2 diabetes mellitus (T2DM), metabolic disorders associated with obesity. *Bacteroides* (SCFA producers) are reported to have a negative correlation with blood pressure while rats with hypertension were found to have decreased quantity of SCFA-producing bacteria (34, 35). While similar observations have been made for pregnant women, it is still unclear how exactly SCFAs contribute to hypertension throughout pregnancy. Similarly, patients with T2DM have decreased concentration of butyrate-producing bacterial species (36). Since butyrate and other SCFAs have been reported to increase insulin sensitivity and glucagon-like-peptide-1 (GLP1) release in animal studies, reduced SCFA levels in pregnant women, and especially pregnant women with obesity, may be contributing factors for the insulin resistance commonly observed during pregnancy (37, 38). Altogether, these studies suggest that the gut microbiota and its byproducts contribute to the metabolic and immune system between women of normal weight and women with obesity.

## 3 Maternal obesity and the infant gut microbiome

### 3.1 Establishment of the infant gut microbiome

The sterile womb hypothesis holds that human infants are born in a sterile environment facilitated by the placental barrier (39). Human studies conducted by Theodore Escherich and Burrage seemed to confirm that neither the amniotic fluid nor placenta contained any bacteria during pregnancy (40). Even recently, researchers confirmed in human studies that the meconium, or the first stool passed by infants, was largely free from any bacteria (41). However, the use of techniques such as quantitative reverse transcription PCR (RT-qPCR) and 16S-rRNA sequencing in human research beginning in the late 20th and early 21st centuries has elucidated findings that challenge the sterile womb hypothesis (42–44). Recent research suggests that in humans, both the fetal lungs and placenta have distinct microbiome profiles as early as the first trimester of pregnancy, and other studies have confirmed that bacterial DNA is present in the human placenta (11, 45). Likewise, the placenta and amniotic fluid have distinct microbiota profiles that share features with the bacteria present in infant meconium, which is characterized by low richness and diversity and high abundance of *Proteobacteria*. Similar findings have been confirmed in animal models, in which mice fetal intestinal bacteria overlaps with the microbiome profile of the placenta (46). In a study of 21 healthy human neonates, researchers found that bacteria of the genera *Enterococcus* and *Staphylococcus* were most prevalent in meconium samples (47). Meconial bacterial composition isolated from the stool of neonates born to healthy mothers both vaginally and by C-section has also been found to differ significantly from the stool of healthy adults, with higher abundance of *Proteobacteria* and lower abundance of *Bacteroidetes* (48). Together, these findings further challenge the sterile womb hypothesis (49). However, the exact route of transmission from the mother’s gut to the placenta and the developing fetus has yet to be clarified.

### 3.2 Effects of maternal obesity on the establishment of the infant gut microbiome

Maternal obesity can significantly alter the composition of the offspring gut microbiome during pregnancy and therefore impact the establishment of the infant’s gut microbiome. Human infants born vaginally to mothers with overweight or obesity had significantly higher levels of *Bacteroides* compared to infants born vaginally to mothers of normal weight (2). Higher species richness within the *Firmicutes* phylum, as well as lower abundance of the *Proteobacteria* families *Enterobacteriaceae* and *Pasteurellaceae* and a higher abundance of the *Firmicutes* family *Lachnospiraceae*, have been reported in infants born to mothers with overweight or obesity (14). These infants were also approximately three times more likely to develop overweight or obesity between 1 and 3 years of age (14). The *Lachnospiraceae* family has been found to be associated with the development of diabetes and adiposity in obese mice (50, 51) however, in humans, *Lachnospiraceae* has shown to be significantly lower in pregnant women with obesity (29). This contradiction illustrates the difficulty of translating animal to human gut microbiota research. Animal studies have demonstrated that high fat diets (HFD) can induce gut dysbiosis, and this obesity-associated gut microbial composition can significantly impact the gut microbial signature of the offspring (52). Fecal transplants from mice fed HFDs cause a reduction in beta-diversity and reduced abundance of *Firmicutes* species when compared to those fed a control diet (52). Female offspring born to the dams that received the stool transplant from HFD mice also had significantly higher body weight and body fat composition 9 weeks after birth when compared to those born to control diet dams, while male offspring had significantly increased anxious and compulsive behavior (52). *Firmicutes* species produce the SCFA butyrate, which has been shown to affect inflammation, behavioral and neurological function, and the maintenance of the gut intestinal barrier in rodents (52–54). It is hypothesized that decreased levels of circulating butyrate may increase the permeability of the intestine and facilitate microbial transfer from the intestinal lumen into the circulation, which could disrupt brain signaling pathways in both humans and rodents (53, 55). Studies in animals have demonstrated that butyrate increases insulin sensitivity while reducing inflammation and food intake; a decrease in butyrate concentration due to both pregnancy and obesity can further influence the maternal and thus the infant’s gut metabolism and immune system (30, 37, 56).

Studies in humans estimate that the odds of developing childhood obesity for infants born to mothers with obesity are between 1.5 and 4 times higher when compared to infants born to mothers of normal weight (2, 14, 57). Research has shown that offspring born to women with obesity had lower levels of fecal butyrate as well as reduced abundance of SCFA-producing bacteria (58). Surprisingly, and in an opposite direction of what we would have expected, researchers also found that the microbiome of infants born to mothers with obesity had greater alpha-diversity at 12 months when compared to infants born to mothers of normal weight, and this alpha-diversity moderately predicted greater adiposity at 12 months of age (58). Thus, it seems that the maternal obesity-associated development of the infant gut microbiome may contribute to the intergenerational transmission of the obesity phenotype (Table 1).

**TABLE 1:** Summary of the impact of human maternal obesity on offspring obesity and gut microbiome colonization.

Finding	References
Infants born to mothers with obesity are 1.5–4 times more likely to develop childhood obesity	(2, 14, 57)
Mothers with obesity are more likely to give birth by C-section, infants born via C-section are more likely to develop childhood obesity	(14, 73)
Delayed gut colonization and ↓ bacterial richness and diversity in C-section delivered infants	(59, 63–66)
↑ *Firmicutes* richness and *Lachnospiraceae* abundance in infants born to mothers with obesity	(14)
Breastfeeding protects against infant gut microbial changes associated with C-section delivery	(88–90)

## 4 Birth delivery methods and the infant gut microbiome

### 4.1 Effects of C-section on the establishment of the infant gut microbiome

In addition to exposure to microbiota in the uterine environment, the colonization of the human infant gut microbiome is also affected by the method of delivery. During vaginal delivery, species of the vagina also contribute to the establishment of the infant intestinal bacteria, and this process has been reported to be disturbed during C-section (59). While a C-section may be critical in reducing infant and maternal mortality in many cases, the differences in gut microbial composition when comparing infants delivered through C-section versus those born vaginally, may have long-term consequences on the offspring’s immune system and metabolism.

Studies in humans have shown that the gut microbiome of infants born via C-section closely resembles that found on the skin (60). This may indicate that unlike infants who are born vaginally, infants born through C-section acquire microbiota through the operating room environment due to the lack of perineal and vaginal contact during delivery (61, 62). Delayed colonization of the gut by species including *Bacteroides*, *Lactobacillus*, *Bifidobacterium*, and *Bacteroidetes* in infants born by Csection has been demonstrated by multiple human studies (63–66). Reyman et al. confirmed that at 1 week, infants born vaginally have significantly higher levels of *Bifidobacterium*, which has shown to be associated with positive health outcomes (67). In a study conducted with 82 newborns, infants born by C-section had significantly higher levels of *Staphylococcus* and *Streptococcus* genera when compared to infants born vaginally, with *Staphylococcus* species remaining significantly greater after 1 month (63). Infants born by C-section were also found to have significantly lower bacterial richness and diversity than infants born vaginally, as well as high levels of *Firmicutes*, especially *Clostridium* and *Enterococcus* (59, 65, 68). Furthermore, researchers have found that bacterial richness, diversity, and composition differ significantly between elective and emergency C-sections (59, 69). Therefore, the mechanism by which these different procedures contribute to the development of the infant gut microbiome needs to be further clarified.

### 4.2 Association between birth delivery and maternal obesity in the transmission of the obesity phenotype

*Staphylococcus* levels are significantly elevated in human infants born by C-section (63). This genus has been shown to be positively associated with obesity and increased energy intake, and as such, it is possible that infants born by C-section may also be at risk for obesity (70–73). Similarly, infants born through C-section have lower α-diversity which has been associated with higher risk to develop obesity and type 2 diabetes (13, 27, 74). Infants born by C-section were between 1.4 and 1.7 times more likely to develop childhood overweight or obesity after adjusting for potential confounders (2, 73, 75, 76).

It should also be noted that maternal overweight and obesity are associated with 1.5 times greater odds of giving birth by C-section, which could in turn mediate the association between maternal and child obesity and thus should be controlled in studies analyzing the gut microbiota (14).

## 5 Discussion

Several factors can be employed to prevent or ameliorate the negative effects brought by maternal obesity and C-section. Probiotic administration during pregnancy, has shown promising results. Particularly, it has shown to increase *Bifidobacterium* abundance in infants (77), and in pre-term infants it has shown to improve intestinal colonization, intestinal barrier, and bacterial diversity, with a reduction of inflammatory cytokines, which would have otherwise contributed to necrotizing enterocolitis, highly common in preterm infants (78, 79). Importantly, however, changes in the microbiome seem to stop after probiotic cessation (80). Thus, the effects of probiotics seem to be short lived.

**FIGURE 1:**
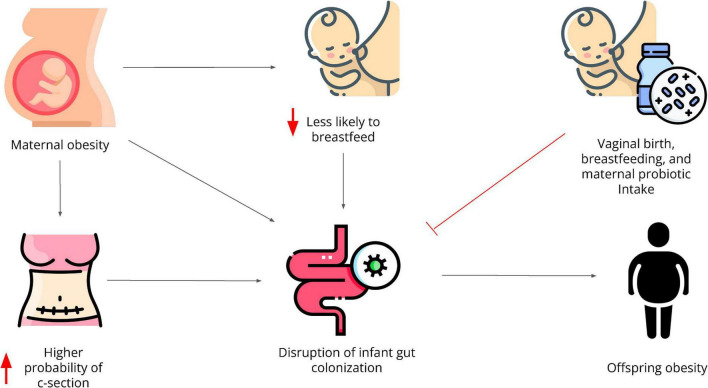
Maternal obesity directly and indirectly contributes to the intergenerational transmission of obesity through disruptions in the gut microbiota. It increases the likelihood to undergoing C-section and it decreases the likelihood of breastfeeding, both of which have independently shown to negatively affect the offspring’s gut microbiome. Infants with an altered gut microbiota are at higher risk to develop obesity amongst many other comorbidities. Vaginal birth, breastfeeding, and probiotic consumption during pregnancy have shown to protect the infant’s gut microbial diversity and composition.

Human breast milk contains many bacterial species that support infant metabolic and immune development (81). Human milk increases microbial diversity, stimulates the growth of beneficial species such as *Bifidobacterium*, and decreases the risk for infections (81–83). *Bifidobacterium longum* in particular, is elevated in human infants that are breastfed, and has been shown to reduce gut permeability (84, 85). Breastfeeding also seems to protect the gut microbiota of babies born through C-section helping them to more closely resemble the gut microbiome of infants born vaginally (86–88). Furthermore, breastfeeding has been shown to decrease the risk of developing obesity and diabetes in childhood and adolescence (89, 90). Unfortunately, pregnant women with obesity, who are more likely to undergo C-section, also show poor breastfeeding practices. Therefore, these practices can individually and in combination reinforce the intergenerational transmission of obesity (Figure 1).

In summary, the gut microbiome may be a key factor in the intergenerational transmission of obesity. Obesity alters a pregnant woman’s gut microbiome’s richness, diversity, composition, and thus derived metabolites, and this microbiota is transferred *in utero* to the infant, which seems to increase their future risks of developing obesity. In addition to maternal obesity, C-section also puts infants at greater risk for developing obesity which is concerning as mothers with obesity are more likely to undergo C-sections and less likely to breastfeed, activity that could help to ameliorate the negative impact of C-section.

To this day, however, identification of the specific gut bacterial species related to the intergenerational transmission of obesity has not been possible. Determining the gut bacterial species and gut-derived metabolites associated with obesity in humans has been difficult due to the high variation in analytical procedures. We encourage future research to study, in addition to gut bacterial composition, the gut bacteria-derived metabolome as the association between these two components can help to better understand the profiles associated with obesity development and transmission. Understanding the gut bacterial composition can help us to identify the direct interactions with the host, whereas analyzing the metabolome can contribute to detect indirect interactions through metabolite production.

## Author contributions

MT conceived the presented idea and wrote the initial draft of the manuscript. EM guided, supervised, and provided critical feedback that helped shape the review to its final form. Both authors contributed to the article and approved the submitted version.

## References

[1] Centers for Disease Control and Prevention [CDC],. *Products - data briefs - number 360 - February 2020 [Internet].* (2020). Available online at: https://www.cdc.gov/nchs/products/databriefs/db360.htm (accessed July, 2022).

[2] Mueller NT Shin H Pizoni A Werlang IC Matte U Goldani MZ Birth mode-dependent association between pre-pregnancy maternal weight status and the neonatal intestinal microbiome. *Sci Rep.* (2016) 6:23133. 10.1038/srep23133 27033998 PMC4817027

[3] Wang Y Lim H. The global childhood obesity epidemic and the association between socioeconomic status and childhood obesity. *Int Rev Psychiatry.* (2012) 24:176–88. 10.3109/09540261.2012.688195 22724639 PMC4561623

[4] World Health Organization [WHO],. *Obesity and overweight [Internet].* (2021). Available online at: https://www.who.int/news-room/fact-sheets/detail/obesity-and-overweight (accessed July, 2022).

[5] Wilson RM Messaoudi I. The impact of maternal obesity during pregnancy on offspring immunity. *Mol Cell Endocrinol.* (2015) 418:134–42. 10.1016/j.mce.2015.07.028 26232506 PMC4674375

[6] O’Reilly JR Reynolds RM. The risk of maternal obesity to the long-term health of the offspring. *Clin Endocrinol.* (2013) 78:9–16. 10.1111/cen.12055 23009645

[7] Zhou L Xiao X. The role of gut microbiota in the effects of maternal obesity during pregnancy on offspring metabolism. *Biosci Rep.* (2018) 38:BSR20171234. 10.1042/BSR20171234 29208770 PMC5897743

[8] Heiss CN Olofsson LE. Gut microbiota-dependent modulation of energy metabolism. *J Innate Immun.* (2018) 10:163–71. 10.1159/000481519 29131106 PMC6757175

[9] Collado MC Isolauri E Laitinen K Salminen S. Distinct composition of gut microbiota during pregnancy in overweight and normal-weight women. *Am J Clin Nutr.* (2008) 88:894–9. 10.1093/ajcn/88.4.894 18842773

[10] Singh SB Madan J Coker M Hoen A Baker ER Karagas MR Does birth mode modify associations of maternal pre-pregnancy BMI and gestational weight gain with the infant gut microbiome? *Int J Obes.* (2020) 44:23–32. 10.1038/s41366-018-0273-0 30765892 PMC6694002

[11] Al Alam D Danopoulos S Grubbs B Ali NABM MacAogain M Chotirmall SH Human fetal lungs harbor a microbiome signature. *Am J Respir Crit Care Med.* (2020) 201:1002–6. 10.1164/rccm.201911-2127LE 31898918 PMC7159424

[12] Sola-Leyva A Andrés-León E Molina NM Terron-Camero LC Plaza-Díaz J Sáez-Lara MJ Mapping the entire functionally active endometrial microbiota. *Hum Reprod.* (2021) 36:1021–31. 10.1093/humrep/deaa372 33598714

[13] Bokulich NA Chung J Battaglia T Henderson N Jay M Li H Antibiotics, birth mode, and diet shape microbiome maturation during early life. *Sci Transl Med.* (2016) 8:343ra82. 10.1126/scitranslmed.aad7121 27306664 PMC5308924

[14] Tun HM Bridgman SL Chari R Field CJ Guttman DS Becker AB Roles of birth mode and infant gut microbiota in intergenerational transmission of overweight and obesity from mother to offspring. *JAMA Pediatr.* (2018) 172:368–77. 10.1001/jamapediatrics.2017.5535 29459942 PMC5875322

[15] Chu SY Kim SY Schmid CH Dietz PM Callaghan WM Lau J Maternal obesity and risk of cesarean delivery: a meta-analysis. *Obes Rev.* (2007) 8:385–94. 10.1111/j.1467-789X.2007.00397.x 17716296

[16] Korpela K Salonen A Vepsäläinen O Suomalainen M Kolmeder C Varjosalo M Probiotic supplementation restores normal microbiota composition and function in antibiotic-treated and in caesarean-born infants. *Microbiome.* (2018) 6:182. 10.1186/s40168-018-0567-4 30326954 PMC6192119

[17] Barbour LA McCurdy CE Hernandez TL Kirwan JP Catalano PM Friedman JE. Cellular mechanisms for insulin resistance in normal pregnancy and gestational diabetes. *Diabetes Care.* (2007) 30 (Suppl. 2):S112–9. 10.2337/dc07-s202 17596458

[18] Catalano PM Tyzbir ED Roman NM Amini SB Sims EAH. Longitudinal changes in insulin release and insulin resistance in nonobese pregnant women. *Am J Obstet Gynecol.* (1991) 165:1667–72. 10.1016/0002-9378(91)90012-G 1750458

[19] Kirwan JP Hauguel-De Mouzon S Lepercq J Challier JC Huston-Presley L Friedman JE TNF-α is a predictor of insulin resistance in human pregnancy. *Diabetes.* (2002) 51:2207–13. 10.2337/diabetes.51.7.2207 12086951

[20] von Versen-Hoeynck FM Powers RW. Maternal-fetal metabolism in normal pregnancy and preeclampsia. *Front Biosci.* (2007) 12:2457–70. 10.2741/2247 17127255

[21] Koren O Goodrich JK Cullender TC Spor A Laitinen K Kling Bäckhed H Host remodeling of the gut microbiome and metabolic changes during pregnancy. *Cell.* (2012) 150:470–80. 10.1016/j.cell.2012.07.008 22863002 PMC3505857

[22] Morgan XC Tickle TL Sokol H Gevers D Devaney KL Ward DV Dysfunction of the intestinal microbiome in inflammatory bowel disease and treatment. *Genome Biol.* (2012) 13:R79. 10.1186/gb-2012-13-9-r79 23013615 PMC3506950

[23] Shin NR Whon TW Bae JW. *Proteobacteria*: microbial signature of dysbiosis in gut microbiota. *Trends Biotechnol.* (2015) 33:496–503. 10.1016/j.tibtech.2015.06.011 26210164

[24] Smid MC Ricks NM Panzer A Mccoy AN Azcarate-Peril MA Keku TO Maternal gut microbiome biodiversity in pregnancy. *Am J Perinatol.* (2018) 35:24–30. 10.1055/s-0037-1604412 28750469 PMC6102712

[25] Nuriel-Ohayon M Neuman H Koren O. Microbial changes during pregnancy, birth, and infancy. *Front Microbiol.* (2016) 7:1031. 10.3389/fmicb.2016.01031 27471494 PMC4943946

[26] DiGiulio DB Callahan BJ McMurdie PJ Costello EK Lyell DJ Robaczewska A Temporal and spatial variation of the human microbiota during pregnancy. *Proc Natl Acad Sci USA.* (2015) 112:11060–5. 10.1073/pnas.1502875112 26283357 PMC4568272

[27] Turnbaugh PJ Ley RE Mahowald MA Magrini V Mardis ER Gordon JI. An obesity-associated gut microbiome with increased capacity for energy harvest. *Nature.* (2006) 444:1027–31. 10.1038/nature05414 17183312

[28] Santacruz A Collado MC García-Valdés L Segura MT Martín-Lagos JA Anjos T Gut microbiota composition is associated with body weight, weight gain and biochemical parameters in pregnant women. *Br J Nutr.* (2010) 104:83–92. 10.1017/S0007114510000176 20205964

[29] Wallace JG Bellissimo CJ Yeo E Fei Xia Y Petrik JJ Surette MG Obesity during pregnancy results in maternal intestinal inflammation, placental hypoxia, and alters fetal glucose metabolism at mid-gestation. *Sci Rep.* (2019) 9:17621. 10.1038/s41598-019-54098-x 31772245 PMC6879619

[30] Zhang M Zhou Q Dorfman RG Huang X Fan T Zhang H Butyrate inhibits interleukin-17 and generates Tregs to ameliorate colorectal colitis in rats. *BMC Gastroenterol.* (2016) 16:84. 10.1186/s12876-016-0500-x 27473867 PMC4967301

[31] Li HP Chen X Li MQ. Butyrate alleviates metabolic impairments and protects pancreatic β cell function in pregnant mice with obesity. *Int J Clin Exp Pathol.* (2013) 6:1574–84. 23923076 PMC3726973

[32] Ziętek M Celewicz Z Szczuko M. Short-chain fatty acids, maternal microbiota and metabolism in pregnancy. *Nutrients.* (2021) 13:1244. 10.3390/nu13041244 33918804 PMC8069164

[33] Szczuko M Kikut J Maciejewska D Kulpa D Celewicz Z Ziętek M. The associations of SCFA with anthropometric parameters and carbohydrate metabolism in pregnant women. *Int J Mol Sci.* (2020) 21:E9212. 10.3390/ijms21239212 33287163 PMC7731050

[34] Soderborg TK Carpenter CM Janssen RC Weir TL Robertson CE Ir D Gestational diabetes is uniquely associated with altered early seeding of the infant gut microbiota. *Front Endocrinol.* (2020) 11:603021. 10.3389/fendo.2020.603021 33329403 PMC7729132

[35] Yang T Santisteban MM Rodriguez V Li E Ahmari N Carvajal JM Gut microbiota dysbiosis is linked to hypertension. *Hypertension.* (2015) 65:1331–40. 10.1161/HYPERTENSIONAHA.115.05315 25870193 PMC4433416

[36] Qin J Li Y Cai Z Li S Zhu J Zhang F A metagenome-wide association study of gut microbiota in type 2 diabetes. *Nature.* (2012) 490:55–60. 10.1038/nature11450 23023125

[37] Tolhurst G Heffron H Lam YS Parker HE Habib AM Diakogiannaki E Short-chain fatty acids stimulate glucagon-like peptide-1 secretion via the g-protein–coupled receptor FFAR2. *Diabetes.* (2012) 61:364–71. 10.2337/db11-1019 22190648 PMC3266401

[38] den Besten G Bleeker A Gerding A van Eunen K Havinga R van Dijk TH Short-chain fatty acids protect against high-fat diet–induced obesity via a PPARγ-dependent switch from lipogenesis to fat oxidation. *Diabetes.* (2015) 64:2398–408. 10.2337/db14-1213 25695945

[39] Funkhouser LJ Bordenstein SR. Mom knows best: the universality of maternal microbial transmission. *PLoS Biol.* (2013) 11:e1001631. 10.1371/journal.pbio.1001631 23976878 PMC3747981

[40] Escherich T. The intestinal bacteria of the neonate and breast-fed infant. 1885. *Rev Infect Dis.* (1989) 11:352–6. 10.1093/clinids/11.2.352 2649968

[41] Hansen R Scott KP Khan S Martin JC Berry SH Stevenson M First-pass meconium samples from healthy term vaginally-delivered neonates: an analysis of the microbiota. *PLoS One.* (2015) 10:e0133320. 10.1371/journal.pone.0133320 26218283 PMC4517813

[42] Dong XD Li XR Luan JJ Liu XF Peng J Luo YY Bacterial communities in neonatal feces are similar to mothers’ placentae. *Can J Infect Dis Med Microbiol.* (2015) 26:90–4. 10.1155/2015/737294 26015791 PMC4419820

[43] Nagpal R Tsuji H Takahashi T Kawashima K Nagata S Nomoto K Sensitive quantitative analysis of the meconium bacterial microbiota in healthy term infants born vaginally or by cesarean section. *Front Microbiol.* (2016) 7:1997. 10.3389/fmicb.2016.01997 28018325 PMC5156933

[44] Aagaard K Ma J Antony KM Ganu R Petrosino J Versalovic J. The placenta harbors a unique microbiome. *Sci Transl Med.* (2014) 6:237ra65. 10.1126/scitranslmed.3008599 24848255 PMC4929217

[45] Satokari R Grönroos T Laitinen K Salminen S Isolauri E. Bifidobacterium and *Lactobacillus* DNA in the human placenta. *Lett Appl Microbiol.* (2009) 48:8–12. 10.1111/j.1472-765X.2008.02475.x 19018955

[46] Martinez KA Romano-Keeler J Zackular JP Moore DJ Brucker RM Hooper C Bacterial DNA is present in the fetal intestine and overlaps with that in the placenta in mice. *PLoS One.* (2018) 13:e0197439. 10.1371/journal.pone.0197439 29771989 PMC5957394

[47] Jiménez E Marín ML Martín R Odriozola JM Olivares M Xaus J Is meconium from healthy newborns actually sterile? *Res Microbiol.* (2008) 159:187–93. 10.1016/j.resmic.2007.12.007 18281199

[48] Hu J Nomura Y Bashir A Fernandez-Hernandez H Itzkowitz S Pei Z Diversified microbiota of meconium is affected by maternal diabetes status. *PLoS One.* (2013) 8:e78257. 10.1371/journal.pone.0078257 24223144 PMC3819383

[49] Perez-Muñoz ME Arrieta MC Ramer-Tait AE Walter J. A critical assessment of the “sterile womb” and “in utero colonization” hypotheses: implications for research on the pioneer infant microbiome. *Microbiome.* (2017) 5:48. 10.1186/s40168-017-0268-4 28454555 PMC5410102

[50] Kameyama K Itoh K. Intestinal colonization by a *Lachnospiraceae* bacterium contributes to the development of diabetes in obese mice. *Microbes Environ.* (2014) 29:427–30. 10.1264/jsme2.ME14054 25283478 PMC4262368

[51] Ravussin Y Koren O Spor A LeDuc C Gutman R Stombaugh J Responses of gut microbiota to diet composition and weight loss in lean and obese mice. *Obesity.* (2012) 20:738–47. 10.1038/oby.2011.111 21593810 PMC3871199

[52] Bruce-Keller AJ Fernandez-Kim SO Townsend RL Kruger C Carmouche R Newman S Maternal obese-type gut microbiota differentially impact cognition, anxiety and compulsive behavior in male and female offspring in mice. *PLoS One.* (2017) 12:e0175577. 10.1371/journal.pone.0175577 28441394 PMC5404786

[53] Stilling RM van de Wouw M Clarke G Stanton C Dinan TG Cryan JF. The neuropharmacology of butyrate: the bread and butter of the microbiota-gut-brain axis? *Neurochem Int.* (2016) 99:110–32. 10.1016/j.neuint.2016.06.011 27346602

[54] Peng L Li ZR Green RS Holzman IR Lin J. Butyrate enhances the intestinal barrier by facilitating tight junction assembly via activation of AMP-activated protein kinase in Caco-2 cell monolayers. *J Nutr.* (2009) 139:1619–25. 10.3945/jn.109.104638 19625695 PMC2728689

[55] Lewis K Lutgendorff F Phan V Söderholm JD Sherman PM McKay DM. Enhanced translocation of bacteria across metabolically stressed epithelia is reduced by butyrate†. *Inflamm Bowel Dis.* (2010) 16:1138–48. 10.1002/ibd.21177 20024905

[56] Li Z Yi CX Katiraei S Kooijman S Zhou E Chung CK Butyrate reduces appetite and activates brown adipose tissue via the gut-brain neural circuit. *Gut.* (2018) 67:1269–79. 10.1136/gutjnl-2017-314050 29101261

[57] Portela DS Vieira TO Matos SM de Oliveira NF Vieira GO. Maternal obesity, environmental factors, cesarean delivery and breastfeeding as determinants of overweight and obesity in children: results from a cohort. *BMC Pregnancy Childbirth.* (2015) 15:94. 10.1186/s12884-015-0518-z 25884808 PMC4407299

[58] Gilley SP Ruebel ML Sims C Zhong Y Turner D Lan RS Associations between maternal obesity and offspring gut microbiome in the first year of life. *Pediatr Obes.* (2022) 17:e12921. 10.1111/ijpo.12921 35478493 PMC9641193

[59] Azad MB Konya T Maughan H Guttman DS Field CJ Chari RS Gut microbiota of healthy Canadian infants: profiles by mode of delivery and infant diet at 4 months. *CMAJ.* (2013) 185:385–94. 10.1503/cmaj.121189 23401405 PMC3602254

[60] Chu DM Ma J Prince AL Antony KM Seferovic MD Aagaard KM. Maturation of the infant microbiome community structure and function across multiple body sites and in relation to mode of delivery. *Nat Med.* (2017) 23:314–26. 10.1038/nm.4272 28112736 PMC5345907

[61] Shin H Pei Z Martinez KA Rivera-Vinas JI Mendez K Cavallin H The first microbial environment of infants born by C-section: the operating room microbes. *Microbiome.* (2015) 3:59. 10.1186/s40168-015-0126-1 26620712 PMC4665759

[62] Shao Y Forster SC Tsaliki E Vervier K Strang A Simpson N Stunted microbiota and opportunistic pathogen colonization in caesarean-section birth. *Nature.* (2019) 574:117–21. 10.1038/s41586-019-1560-1 31534227 PMC6894937

[63] Pan K Zhang C Tian J. The effects of different modes of delivery on the structure and predicted function of intestinal microbiota in neonates and early infants. *Pol J Microbiol.* (2021) 70:45–55. 10.33073/pjm-2021-002 33815526 PMC8008759

[64] Dogra S Sakwinska O Soh SE Ngom-Bru C Brück WM Berger B Dynamics of infant gut microbiota are influenced by delivery mode and gestational duration and are associated with subsequent adiposity. *mBio.* (2015) 6:e02419-14. 10.1128/mBio.02419-14 25650398 PMC4323417

[65] Jakobsson HE Abrahamsson TR Jenmalm MC Harris K Quince C Jernberg C Decreased gut microbiota diversity, delayed Bacteroidetes colonisation and reduced Th1 responses in infants delivered by Caesarean section. *Gut.* (2014) 63:559–66. 10.1136/gutjnl-2012-303249 23926244

[66] Biasucci G Rubini M Riboni S Morelli L Bessi E Retetangos C. Mode of delivery affects the bacterial community in the newborn gut. *Early Hum Dev.* (2010) 86:13–5. 10.1016/j.earlhumdev.2010.01.004 20133091

[67] Reyman M van Houten MA van Baarle D Bosch AATM Man WH Chu MLJN Impact of delivery mode-associated gut microbiota dynamics on health in the first year of life. *Nat Commun.* (2019) 10:4997. 10.1038/s41467-019-13373-1 31676793 PMC6825150

[68] Murata C Gutiérrez-Castrellón P Pérez-Villatoro F García-Torres I Enríquez-Flores S de la Mora-de la Mora I Delivery mode-associated gut microbiota in the first 3 months of life in a country with high obesity rates: a descriptive study. *Medicine.* (2020) 99:e22442. 10.1097/MD.0000000000022442 33019428 PMC7535699

[69] Hoang DM Levy EI Vandenplas Y. The impact of Caesarean section on the infant gut microbiome. *Acta Paediatr.* (2021) 110:60–7. 10.1111/apa.15501 33405258

[70] Collado MC Isolauri E Laitinen K Salminen S. Effect of mother’s weight on infant’s microbiota acquisition, composition, and activity during early infancy: a prospective follow-up study initiated in early pregnancy. *Am J Clin Nutr.* (2010) 92:1023–30. 10.3945/ajcn.2010.29877 20844065

[71] Bervoets L Van Hoorenbeeck K Kortleven I Van Noten C Hens N Vael C Differences in gut microbiota composition between obese and lean children: a cross-sectional study. *Gut Pathog.* (2013) 5:10. 10.1186/1757-4749-5-10 23631345 PMC3658928

[72] Gomes AC Hoffmann C Mota JF. The human gut microbiota: metabolism and perspective in obesity. *Gut Microbes.* (2018) 9:308–25. 10.1080/19490976.2018.1465157 29667480 PMC6219651

[73] Sitarik AR Havstad SL Johnson CC Jones K Levin AM Lynch SV Association between cesarean delivery types and obesity in preadolescence. *Int J Obes.* (2020) 44:2023–34. 10.1038/s41366-020-00663-8 32873910 PMC7530127

[74] Kostic AD Gevers D Siljander H Vatanen T Hyötyläinen T Hämäläinen AM The dynamics of the human infant gut microbiome in development and in progression toward type 1 diabetes. *Cell Host Microbe.* (2015) 17:260–73. 10.1016/j.chom.2015.01.001 25662751 PMC4689191

[75] Li HT Zhou YB Liu JM. The impact of cesarean section on offspring overweight and obesity: a systematic review and meta-analysis. *Int J Obes.* (2013) 37:893–9. 10.1038/ijo.2012.195 23207407

[76] Pei Z Heinrich J Fuertes E Flexeder C Hoffmann B Lehmann I Cesarean delivery and risk of childhood obesity. *J Pediatr.* (2014) 164:1068–73.e2. 10.1016/j.jpeds.2013.12.044 24508442

[77] Gueimonde M Sakata S Kalliomäki M Isolauri E Benno Y Salminen S. Effect of maternal consumption of lactobacillus GG on transfer and establishment of fecal bifidobacterial microbiota in neonates. *J Pediatr Gastroenterol Nutr.* (2006) 42:166–70. 16456409 10.1097/01.mpg.0000189346.25172.fd

[78] Kapourchali FR Cresci GAM. Early-life gut microbiome—the importance of maternal and infant factors in its establishment. *Nutr Clin Pract.* (2020) 35:386–405. 10.1002/ncp.10490 32329544

[79] Wang Q Dong J Zhu Y. Probiotic supplement reduces risk of necrotizing enterocolitis and mortality in preterm very low-birth-weight infants: an updated meta-analysis of 20 randomized, controlled trials. *J 1Pediatr Surg.* (2012) 47:241–8. 10.1016/j.jpedsurg.2011.09.064 22244424

[80] Martín-Peláez S Cano-Ibáñez N Pinto-Gallardo M Amezcua-Prieto C. The impact of probiotics, prebiotics, and synbiotics during pregnancy or lactation on the intestinal microbiota of children born by cesarean section: a systematic review. *Nutrients.* (2022) 14:341. 10.3390/nu14020341 35057522 PMC8778982

[81] Lyons KE Ryan CA Dempsey EM Ross RP Stanton C. Breast milk, a source of beneficial microbes and associated benefits for infant health. *Nutrients.* (2020) 12:E1039. 10.3390/nu12041039 32283875 PMC7231147

[82] Morozov V Hansman G Hanisch FG Schroten H Kunz C. Human milk oligosaccharides as promising antivirals. *Mol Nutr Food Res.* (2018) 62:1700679. 10.1002/mnfr.201700679 29336526

[83] Lee SA Lim JY Kim BS Cho SJ Kim NY Kim OB Comparison of the gut microbiota profile in breast-fed and formula-fed Korean infants using pyrosequencing. *Nutr Res Pract.* (2015) 9:242–8. 10.4162/nrp.2015.9.3.242 26060535 PMC4460055

[84] Chichlowski M De Lartigue G German JB Raybould HE Mills DA. Bifidobacteria isolated from infants and cultured on human milk oligosaccharides affect intestinal epithelial function. *J Pediatr Gastroenterol Nutr.* (2012) 55:321–7. 10.1097/MPG.0b013e31824fb899 22383026 PMC3381975

[85] Wickramasinghe S Pacheco AR Lemay DG Mills DA. Bifidobacteria grown on human milk oligosaccharides downregulate the expression of inflammation-related genes in Caco-2 cells. *BMC Microbiol.* (2015) 15:172. 10.1186/s12866-015-0508-3 26303932 PMC4548914

[86] Princisval L Rebelo F Williams BL Coimbra AC Crovesy L Ferreira AL Association between the mode of delivery and infant gut microbiota composition up to 6 months of age: a systematic literature review considering the role of breastfeeding. *Nutr Rev.* (2021) 80:113–27. 10.1093/nutrit/nuab008 33837424

[87] Guo C Zhou Q Li M Zhou L Xu L Zhang Y Breastfeeding restored the gut microbiota in caesarean section infants and lowered the infection risk in early life. *BMC Pediatr.* (2020) 20:532. 10.1186/s12887-020-02433-x 33238955 PMC7690020

[88] Liu Y Qin S Song Y Feng Y Lv N Xue Y The perturbation of infant gut microbiota caused by cesarean delivery is partially restored by exclusive breastfeeding. *Front Microbiol.* (2019) 10:598. 10.3389/fmicb.2019.00598 30972048 PMC6443713

[89] Ma J Qiao Y Zhao P Li W Katzmarzyk PT Chaput J Breastfeeding and childhood obesity: a 12-country study. *Matern Child Nutr.* (2020) 16:e12984. 10.1111/mcn.12984 32141229 PMC7296809

[90] Ortega-García JA Kloosterman N Alvarez L Tobarra-Sánchez E Cárceles-Álvarez A Pastor-Valero R Full breastfeeding and obesity in children: a prospective study from birth to 6 years. *Child Obes.* (2018) 14:327–37. 10.1089/chi.2017.0335 29912590 PMC6066191

